# Population differentiation, antifungal susceptibility, and host range of *Trichophyton mentagrophytes* isolates causing recalcitrant infections in humans and animals

**DOI:** 10.1007/s10096-020-03952-2

**Published:** 2020-06-30

**Authors:** Sebastian Gnat, Dominik Łagowski, Aneta Nowakiewicz, Marcelina Osińska, Łukasz Kopiński

**Affiliations:** 1 Faculty of Veterinary Medicine, Institute of Biological Bases of Animal Diseases, Department of Veterinary Microbiology, University of Life Sciences, Akademicka 12, 20-033 Lublin, Poland; 2 Faculty of Agrobioengineering, Department of Management and Marketing, University of Life Sciences, Dobrzanskiego 37, 20-626 Lublin, Poland

**Keywords:** Antifungal resistance, Dermatophytes, Genomic diversity, Pathogenicity, *Trichophyton mentagrophytes*, Therapy, Public health, Keratinolytic activity

## Abstract

The major problems in determining the causative factors of the high prevalence of dermatophytoses include the lack of a well-standardized antifungal susceptibility testing method, the low consistency of in vitro and clinical minimal inhibitory concentration values, the high genomic diversity of the population, and the unclear mechanism of pathogenicity. These factors are of particular importance when the disease is recalcitrant and relapses. Herein, we identified and characterized *Trichophyton mentagrophytes* isolates obtained from therapy-resistant cases in humans and animals. We used genomic diversity analysis of 17 human and 27 animal clinical isolates with the MP-PCR technique, determined their phenotypic enzymatic activity and host range, and performed antifungal susceptibility testing to currently available antifungal drugs from various chemical groups. Genomic diversity values of 35.3% and 33.3% were obtained for clinical isolates from humans and animals, respectively, yet without any relationship to the host species or antifungal drug to which resistance in therapy was revealed. The highest activity of keratinase enzymes was recorded for fox, guinea pig, and human hairs. These hosts can be considered as the main species in the host range of these isolates. A phenyl morpholine derivative, i.e. amorolfine, exhibited superior activity against strains obtained from both humans and animals with the lowest MIC_50_. Interestingly, high compliance of terbinafine in vitro resistance with clinical problems in the treatment with this substance was shown as well. The high resistance of dermatophytes to drugs is the main cause of the recalcitrance of the infection, whereas the other features of the fungus are less important.

## Introduction

Dermatophytes are the most commonly encountered fungi in humans and other vertebrates spreading through direct or indirect contacts with infected individuals and soil [1, 2]. Epidemiological studies have documented a varied prevalence rate of dermatophytosis ranging from 14 to 26.8% in North America, Asia, and Europe and from 5 to 31.6% in Africa (Ethiopia, Kenya, Nigeria, and Tanzania) [3–7]. An alarming upward trend in the incidence of superficial dermatophytosis has been especially noticed in Europe and Asia over the past 5–10 years [8, 9]. Although the high prevalence of dermatophytosis is a consequence of climate change and new living habits of society, a dramatic change in the clinical features of patients is also noted, as these infections are characterized by recalcitrant response to treatment and increasing relapse rates [10, 11]. The cause of this phenomenon is not yet clear.

Considering the enormous number of taxonomic differences between dermatophytes that can be tested and the importance of species-level identification, the “gold standard” to use for routine mycological identification has still become the topic of a debate, and no uniform position of microbiologists has been developed [8, 12, 13]. Nonetheless, the advent of molecular methods in mycology facilitates identification of dermatophytes to the species level in a rapid and accurate manner [14–16]. However, other major problems remain, i.e. the lack of a well-standardized antifungal susceptibility testing method and the low consistency of in vitro and clinical minimal inhibitory concentration values [16–21]. In addition, although many studies of the mechanism of the pathogenicity of dermatophytes have been carried out over the years, there have been no concrete proposals whether it is possible to construct a profile of animal hosts susceptible to individual species of dermatophytes [22–25]. In this context, it is difficult to clearly determine whether the growing prevalence of dermatophytoses is caused only by changes observed in the natural environment and lifestyles or also by increased host sensitivity, a higher degree of dermatophyte pathogenicity, or the weakness of the currently available antifungal arsenal [18, 19, 22, 26].

Recent studies have demonstrated emerging predominance of members of the *Trichophyton mentagrophytes* species complex as the causative organisms in many cases of dermatophytoses [9, 18, 27–30]. *Trichophyton mentagrophytes* is primarily a zoophilic dermatophyte which often attacks humans through direct or indirect transmission from animals and can rarely survive saprophytically in the soil [1, 2]. Infections caused by this species have been reported in a large number of wild and domestic animals including pets (guinea pigs, hamsters, rabbits, chinchillas) and fur animals (foxes, ferrets, wolf, mink) [29, 31, 32]. Interestingly, zoophilic fungal infections caused by *T. mentagrophytes* commonly occur especially in 3–7-year-old children and the elderly through purchase of asymptomatic pet carriers in zoological shops [33, 34].

Herein, we identified and investigated recalcitrant *T. mentagrophytes* infections in humans and animals. The aim of this study was to analyse the clinical isolates of dermatophytes in terms of their genomic diversity, phenotypic degree of pathogenicity, and in vitro susceptibility to antifungal drugs.

## Materials and methods

### Patient details and identification

**Fig. 1:**
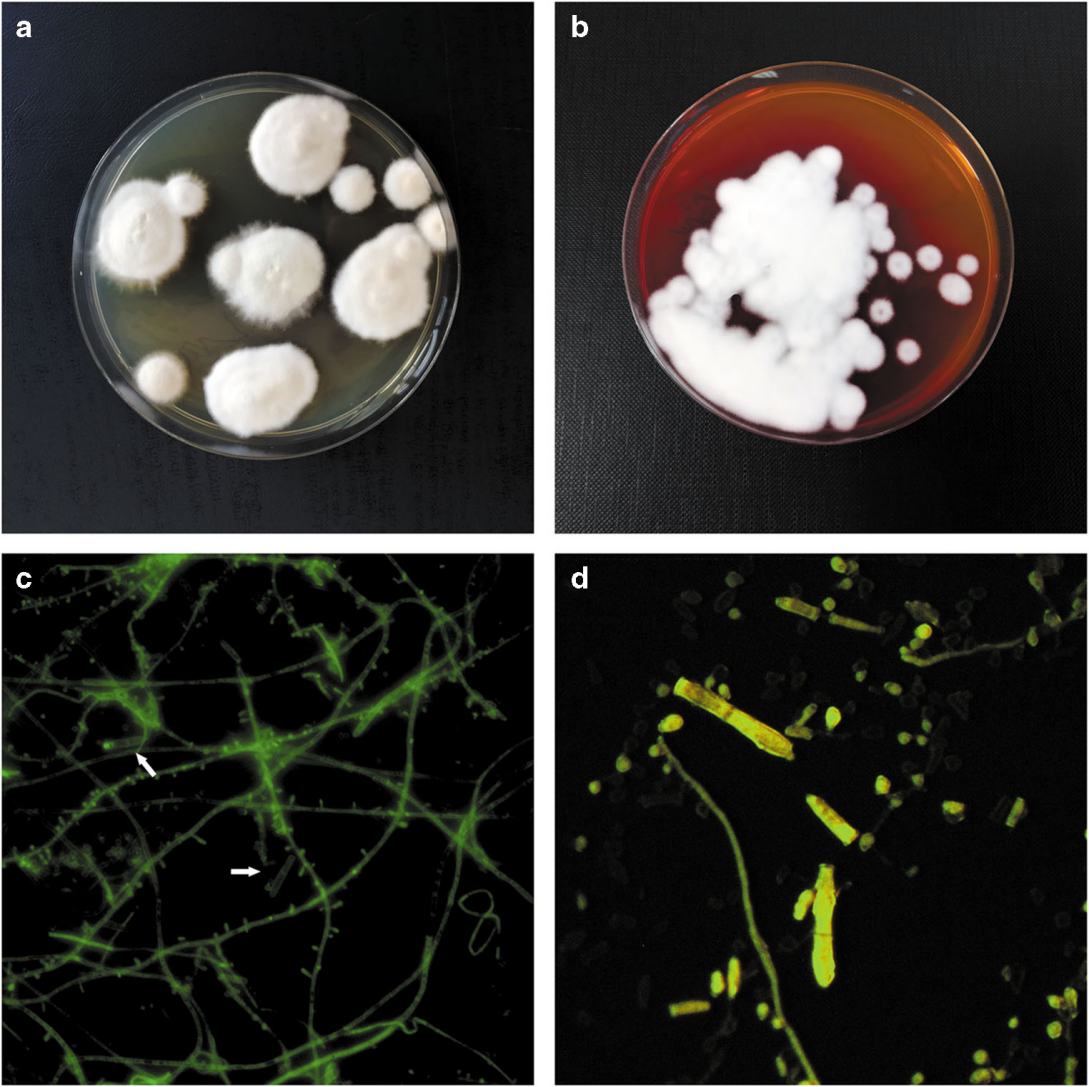
Micro- and macroscopic morphology of *Trichophyton mentagrophytes* strains after 14 days of incubation. Notes: Colonies flat, white in colour, with a powdery surface. The size of the colony in the range from 10 to 15 mm. The edges of the colony are smooth with a slight furrow in the form of a star. Image from Sabouraud medium (**a**) and from DTM (dermatophyte test medium) (**b**). Numerous single-celled microconidia are formed, often in dense clusters. The micromorphological image (taken with a fluorescence microscope Olympus BX51) on the microscope slide stained with calcofluor white revealed numerous hyaline, smooth-walled, and spherical microconidia placed laterally on hyphae (**c**, magnification ×400). The multicelled, cigar-shaped macroconidia are sporadic (**d**, magnification ×1000)

Dermatophytes were detected from skin scrapings and hairs of 24 humans and 35 animals who had previously received oral and topical treatment for a period of at least 28 days (Table 1). None of the patients at that time took any other medications or received immunosuppressive therapy. Cases of infection were diagnosed between 2018 and 2019 in Poland. Tinea capitis: *n* = 15 (62.5%) was the predominant clinical form in the human infections followed by tinea corporis: *n* = 7 (29.2%) and tinea unguium: *n* = 2 (8.3%). In the animals, ringworm located around the head and neck: *n* = 17 (48.6%), on the torso: *n* = 8 (22.9%), and in multiple sites on the body: *n* = 9 (25.7%) was reported. In turn, dermatophytes were isolated from 17 (70.8%) human and 27 (77.1%) animal samples that were positive in the real-time PCR tests. The sample was collected from lesion margins. Detection and identification of dermatophytes in the dermatological material from the patients was conducted using the real-time PCR assay according to Ohst et al. [35]. Reactions with pan-dermatophyte primers (F: 5′-AGCGCYCGCCGRAGGA-3′; R: 5′-GATTCACGGAATTCTGCAATTCAC-3′) and species-specific primers (F: 5′-CGGCGAGCCTCTCTTTAGT-3′; R: 5′-GATTCACGGAATTCTGCAATTCAC-3′) targeting ITS1, ITS2, and 5.8S rDNA gene sequences in combination with TaqMan probes (Derm5.8S: CGCATTTCGCTGCGTTCTTCATC) were made to identify clinical isolates. DNA isolation and real-time PCR were carried out using a DNeasy Blood & Tissue Kit and a Quanti Tect SYBR green PCR master mix (Qiagen, Hilden, Germany), respectively. A samples with dermatophyte identified in the real-time technique were directed to isolation of cultures. The species identification for full confirmation of the taxonomic position was based on macro- and microscopic examination according to de Hoog et al. [36] (Fig. 1).

**Table 1:** Samples tested and isolates of *Trichophyton mentagrophytes* obtained from animals and humans with a description

Isolates	Host	Isolation source	Oral treatment	Topical treatment	Duration (days)	Real-time PCR identification		Culture
						Pan-dermatophyte	Species-specific	
TMH1/20	Human	Tinea capitis	Terbinafine	Ciclopirox	32	+	*+*	*+*
TMH2/20	Human	Tinea capitis	Terbinafine	Ketoconazole	28	+	−	−
TMH3/20	Human	Tinea capitis	Terbinafine	Ketoconazole	34	+	*+*	*+*
TMH4/20	Human	Tinea capitis	Terbinafine	Ketoconazole	34	+	*+*	*+*
TMH5/20	Human	Tinea capitis	Ketoconazole	Naftifine	30	+	−	−
TMH6/20	Human	Tinea corporis	Ketoconazole	Ciclopirox	28	+	−	−
TMH7/20	Human	Tinea unguium	Fluconazole	Amorolfine	44	+	*+*	*+*
TMH8/20	Human	Tinea capitis	Terbinafine	Ciclopirox	28	+	*+*	*+*
TMH9/20	Human	Tinea corporis	Itraconazole	Ciclopirox	29	+	*+*	*+*
TMH10/20	Human	Tinea corporis	Itraconazole	Ciclopirox	28	+	*+*	*+*
TMH1/19	Human	Tinea capitis	Ketoconazole	Terbinafine	36	+	*+*	*+*
TMH2/19	Human	Tinea capitis	Terbinafine	Ketoconazole	29	+	*+*	−
TMH3/19	Human	Tinea corporis	Itraconazole	Ciclopirox	38	+	*+*	*+*
TMH4/19	Human	Tinea unguium	Fluconazole	Amorolfine	51	+	*+*	*+*
TMH5/19	Human	Tinea capitis	Terbinafine	Ciclopirox	37	+	*+*	*+*
TMH6/19	Human	Tinea capitis	Terbinafine	Ketoconazole	28	+	*+*	*+*
TMH7/19	Human	Tinea capitis	Terbinafine	Ketoconazole	28	+	*+*	*+*
TMH8/19	Human	Tinea corporis	Itraconazole	Naftifine	29	+	*+*	−
TMH9/19	Human	Tinea corporis	Itraconazole	Naftifine	32	+	−	−
TMH10/19	Human	Tinea capitis	Ketoconazole	Naftifine	28	+	*+*	*+*
TMH11/19	Human	Tinea capitis	Ketoconazole	Naftifine	28	+	*+*	*+*
TMH12/19	Human	Tinea capitis	Terbinafine	Ciclopirox	36	+	*+*	*+*
TMH13/19	Human	Tinea capitis	Terbinafine	Ketoconazole	41	+	*+*	*+*
TMH14/19	Human	Tinea corporis	Itraconazole	Terbinafine	36	+	−	−
TMA1/20	Fox	Head	–	Enilconazole	37	+	−	−
TMA2/20	Fox	Head	–	Enilconazole	37	+	−	−
TMA3/20	Fox	Head, neck	–	Enilconazole	37	+	*+*	*+*
TMA4/20	Fox	Neck	–	Enilconazole	45	+	*+*	*+*
TMA5/20	Fox	Torso	–	Enilconazole	45	+	*+*	*+*
TMA6/20	Fox	Multiple	–	Enilconazole	28	+	*+*	*+*
TMA7/20	Fox	Multiple	–	Enilconazole	28	+	*+*	*+*
TMA8/20	Fox	Head	–	Enilconazole	28	+	*+*	*+*
TMA9/20	Fox	Neck	–	Enilconazole	28	+	*+*	*+*
TMA10/20	Fox	Torso	–	Enilconazole	28	+	*+*	*+*
TMA11/20	Guinea pig	Head	Terbinafine	Miconazole	31	+	−	−
TMA12/20	Guinea pig	Head, neck	Terbinafine	Miconazole	29	+	*+*	−
TMA13/20	Guinea pig	Torso	Itraconazole	–	37	+	*+*	*+*
TMA14/20	Guinea pig	Multiple	Griseofulvin	Lime sulphur	46	+	*+*	*+*
TMA15/20	Guinea pig	Multiple	Griseofulvin	Lime sulphur	46	+	*+*	*+*
TMA16/20	Guinea pig	Multiple	Itraconazole	–	32	+	*+*	*+*
TMA1/19	Rabbit	Head	Itraconazole	–	32	+	*+*	*+*
TMA2/19	Rabbit	Torso	Itraconazole	–	32	+	−	−
TMA3/19	Fox	Head	–	Enilconazole	49	+	−	−
TMA4/19	Fox	Multiple	–	Enilconazole	49	+	*+*	*+*
TMA5/19	Fox	Torso	–	Enilconazole	28	+	*+*	*+*
TMA6/19	Guinea pig	Head	Itraconazole	Miconazole	44	+	*+*	*+*
TMA7/19	Guinea pig	Neck	Itraconazole	Miconazole	34	+	*+*	*+*
TMA8/19	Fox	Torso	–	Enilconazole	58	+	*+*	*+*
TMA9/19	Cat	Head, neck	Itraconazole	–*	28	+	*+*	*+*
TMA10/19	Cat	Torso	Itraconazole	–*	28	+	*+*	*+*
TMA11/19	Dog	Head	Itraconazole	–*	28	+	−	−
TMA12/19	Dog	Neck	Itraconazole	–*	33	+	*+*	*+*
TMA13/19	Dog	Multiple	Griseofulvin	Miconazole	45	+	*+*	*+*
TMA14/19	Fox	Head, neck	–	Enilconazole	26	+	*+*	*+*
TMA15/19	Fox	Torso	–	Enilconazole	31	+	*+*	*+*
TMA16/19	Guinea pig	Multiple	Griseofulvin	Lime sulphur	46	+	*+*	*+*
TMA17/19	Rabbit	Head	Terbinafine	Miconazole	29	+	−	−
TMA18/19	Rabbit	Head, neck	Terbinafine	Miconazole	28	+	*+*	*+*
TMA19/19	Rabbit	Multiple	Griseofulvin	Lime sulphur	42	+	*+*	*+*

*Vaccine used instead of topical treatment

### Genetic diversity of dermatophyte isolates

The genetic diversity of the clinical isolates was performed with the melting point PCR (MP-PCR) method optimized and modified for dermatophytes [31]. Briefly, the first step was the digestion of the genomic DNA isolated from the cultures (phenol-chloroform method [37]) using *Hind*III (Thermo Fisher, Waltham, USA) endonuclease. Next, an adaptor, i.e. a mixture of two oligonucleotides Helper and Ligated (Helper: 5′-AGCTGTCGACGTTGG-3′, Ligated: 5′-CTCACTCTCACCAACAACGTCGAC-3′), was ligated to DNA fragments. The PCR was made with a primer terminated with the AGCTT adapter sequence (PowaAGCT 5′-CTCACTCTCACCAACGTCGACAGCTT-3′). Electrophoresis of all PCR products was carried out in 3% agarose gels. *T. mentagrophytes* CBS570.80 and CBS677.86 were used as the reference strains. All analyses were made in triplicate.

### Evaluation of production of virulence factors

The production of virulence factors was evaluated using specific test media. The following tests were performed: production of keratinase (1), elastase (2), phospholipase (3), lipase (4), protease (5), gelatinase (6), and detection of haemolytic activity (7). Keratinase production (1): the medium was prepared as described by Scott and Untereiner [38]. The basal layer containing 0.5 g/l MgSO_4_·7H_2_O, 0.05 g/l KCl, 0.5 g/l K_2_HPO_4_, 0.1 g/l ZnSO_4_·7H_2_O, 0.1 g/l FeSO_4_·7H_2_O, 0.03 g/l CuSO_4_, and 25.0 g/l agar was dispensed horizontally in sterilized test tubes. The upper layer was supplemented with 4 mg/ml keratin azure. Clinical isolates inoculated with the medium grew for 1 month in the dark at 37 °C. A change of the colour of the basal layer from milky white to blue was a positive result. *Chrysosporium keratinophilum* CBS104.62 was used as a positive control. The elastin activity (2) was determined on two-layer plates as described by Rippon and Varadi [39]. The basal layer containing 8.0 g/l nutrient broth and 20.0 g/l Agar Noble was poured in a thin layer onto Petri dishes; the upper layer was supplemented with 3.4 g/l elastin from bovine neck ligament. Brighter zones around the colonies after incubation at 37 °C for 14 days indicated elastinase activity. *Pseudomonas aeruginosa* ATCC15152 was used as a positive control. The phospholipase assay (3) was performed according to the method described by Gnat et al. [40]. Briefly, the medium containing (g/l) 10.0 g/l peptone, 20.0 g/l dextrose, 57.3 g/l NaCl, 0.005 g/l CaCl_2_, 20.0 g/l agar, and 50 ml egg yolk was punctiform inoculated with a single dermatophyte colony in the centre of the plate and incubated for 14 days at 28 °C. A clear halo zone around the colony indicated phospholipase production. *Candida albicans* ATCC10231 was used as a positive control. The lipase activity (4) was tested on medium containing 10.0 g/l peptone, 5.0 g/l NaCl, 0.1 g/l CaCl_2_, 20.0 g/l agar, and 10 ml Tween 80 as described by Muhsin et al. [41]. Isolates of dermatophytes were punctiform inoculated in the centre of the plate and incubated for 14 days at 28 °C. A clear halo zone of precipitation around the colony indicated lipase production. *Malassezia furfur* ATCC14521 was used as a positive control. Protease activity (5) was tested on casein medium with bromocresol green (BCG) as described by Vijayaraghavan et al. [42]. The medium containing 5.0 g/l meat peptone 1.5 g/l beef extract, 1.5 g/l yeast extract, 5.0 g/l NaCl, agar, 15, 10.0 g/l casein, 0.0015% (w/v) BCG, and 15.0 g/l agar was punctiform inoculated in the centre of the plate and incubated for 14 days at 28 °C. A zone of proteolysis around the colony was a positive result. *Bacillus subtilis* ATCC6633 was used as a control. The gelatinase assay (6) was performed according to the method described by Gnat et al. [40]. The medium containing 5.0 g/l neopeptone, 3.0 g/l beef extract, and 120.0 g/l gelatin was poured into tubes, inoculated, and incubated at 25 °C for a month. After this period, all tubes were cooled to 4 °C and tilted to check if the gelatin was liquefied. The gelatinase secretion was determined on the basis of the physical state of the medium: liquid medium was a positive result. *Staphylococcus aureus* ATCC29213 was used as a positive control. The haemolytic activity (7) was assessed using Columbia agar medium (BioMaxima, Lublin, Poland) supplemented with 5% defibrated sheep blood as described by Schaufuss et al. [43]. A needle was used for punctiform inoculation with a single colony, and then incubation at 28 °C lasted for 7 days. The presence of a translucent halo around the colony was a positive result. *Staphylococcus aureus* ATCC25923 was used as a positive control for β-haemolysis, *Streptococcus pneumoniae* ATCC6305 for α-haemolysis, and *Clostridium perfringens* ACTCC13124 for double zone haemolysis. The catalase activity was determined on medium containing 3% hydrogen peroxide and 2% agar. Wells cut out on a Petri dish were supplemented with the dermatophyte suspension. The plates were incubated at 28 °C for 6 h aseptically. The staining solution containing 2% of K_3_Fe(CN)_6_ and FeCl_3_·6H_2_O each was prepared ex tempore. The staining solution was poured into Petri dishes containing the samples, which were then shaken gradually until a green colour appeared. The staining solution was then filtered off, and the plate was rinsed and filled with distilled water. The appearance of a yellow ring around the well was considered a positive result. *Staphylococcus aureus* ATCC6538 was used as a positive control. For each test, the clinical isolates of the dermatophytes were incubated on MM-Cove medium with keratin azure (Sigma-Aldrich, Missouri, USA) for 14 days at 28 °C. Unless otherwise described, a halo zone diameter exceeding 50% of the dermatophyte colony diameter was regarded as a positive result. Each test was made in triplicate.

### Determining the host range of isolates

The host range of the dermatophytes was determined with the method described previously by Gnat et al. [44]. The basis for the determination was the diverse keratinolytic activity against species-specific keratin substrates. Cultures used for this test were inoculated onto liquid Sabouraud glucose agar (BioMaxima, Lublin, Poland) at 37 °C for 15 days with shaking at 110 rpm (Multitron, Infors, Switzerland). Next, the mycelium was collected and mechanically homogenized, resuspended to a concentration of 1 in McFarland, and used as the source of enzymes. Hairs and fur obtained from *Bos taurus* (cow), *Canis familiaris* (dog), *Cavia porcellus* (guinea pig), *Equus caballus* (horse), *Felis catus* (cat), *Homo sapiens* (human), *Ovis aries* (sheep), *Sus domesticus* (pig), and *Vulpes vulpes* (fox) during routine hygiene operations were cut separately into 1-mm pieces, washed, and defatted. After drying, they were used as substrates. Keratin azure (Sigma-Aldrich, Missouri, USA) was used as a positive control and indicator substrate. The substrates and sources of the enzyme were added to a liquid minimal medium (MM-Cove) designed to determine keratinolytic activity at a concentration of 0.06% and in a volume 100 μl, respectively. The enzymatic activity (Uh^−1^) was determined spectrophotometrically at a wavelength of 550 nm (SmartSpec, BioRad, USA) as mycelium growth per hour of incubation on incubation days 4, 7, 10, 15, 30, and 60. The test was performed in three replicates simultaneously, and differences between enzymatic activity in different periods of analysis as well as various substrates were assessed by Student’s *t* test using the R program version 3.6.3 (R Core Team, Missouri, USA).

### Antifungal susceptibility tests

In vitro testing of the susceptibility to allylamine, polyene, imidazole, triazole, and pyridinone derivatives as well as phenyl morpholine derivatives was performed according to the Clinical and Laboratory Standards Institute (CLSI) document M38-A3 (CLSI, 2018). Reagent-grade amorolfine (AMR), amphotericin B (AMB), ciclopirox (CPO), enilconazole (ENC), fluconazole (FLC), griseofulvin (GRE), itraconazole (ITC), ketoconazole (KTC), miconazole (MCZ), naftifine (NFT), terbinafine (TRB), and voriconazole (VRC) were obtained in the powder form (Sigma-Aldrich, Missouri, USA). Drug stock solutions were prepared in dimethyl sulfoxide (DMSO) to reach the final DMSO concentration in the wells below 1%. The drugs were analysed at the final concentration in the range of 0.002–2 μg/ml for allylamine, pyridinone derivatives, and phenyl morpholine derivatives, 0.004–4 μg/ml for polyenes, imidazoles, itraconazole, and voriconazole, and 0.06–64 μg/ml for fluconazole. The dermatophyte isolates were cultured on Sabouraud glucose agar (BioMaxima, Lublin, Poland) for 21 days, and conidial suspensions were prepared by gentle scraping mature colonies into sterile physiological saline containing 0.002% Tween 80. Homogeneous inoculum supernatants were collected, and their optical density (OD) at 530 nm was adjusted spectrophotometrically to an OD of 0.11 to 0.13, which ranged from 65 to 70% transmission, and the final density of inoculum was 1 × 10^3^ to 3 × 10^3^ CFU/ml. The inocula were diluted 1:50 in RPMI 1640 medium and incubated with the indicated concentrations of the antifungals in 96-well plates at 35 °C for 72 h. Minimum inhibitory concentrations (MICs) were determined visually using a reading mirror as complete inhibition of observable growth. *Trichophyton mentagrophytes* ATCC4439 and *T. rubrum* ATCC4438 served as quality controls for every new series of MIC plates. All tests were performed in triplicate, and differences between mean values were assessed by Student’s *t* test using the R program version 3.6.3 (R Core Team, Missouri, USA).

## Results

### Genetic diversity of dermatophyte isolates

**Fig. 2:**
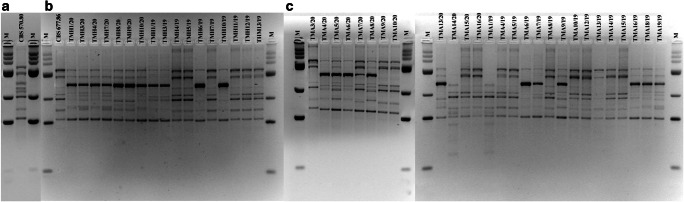
Electrophoretic profile obtained by Melting Profile (MP-PCR) in 3% agarose gel. Notes: **a** reference strain *Trichophyton mentagrophytes* CBS570.80, **b** reference strain *Trichophyton mentagrophytes* complex CBS677.86 and isolates from humans, **c** isolates from animals. In the first line M – molecular weight marker GeneRuler™ 100 bp DNA Ladder Plus (100–3000 bp; Thermo Fisher, Waltham, USA)

In this study, the dermatophytes isolated from human and animal infections in Poland were characterized for genomic diversity and relationships with the commonly used DNA fingerprinting technique based on PCR reaction, i.e. MP-PCR. Six genotypes of the 17 human isolates of *T. mentagrophytes* were distinguished (Fig. 2). In turn, nine genotypes were identified in the animal isolates. In each case, similar genomic diversity values of 35.3% and 33.3% were obtained for the clinical isolates from humans and animals, respectively. No relationship was demonstrated between the antifungal drug resistance profile and the MP-PCR profile. In addition, no relationship between the profile and the host species was found for the animal isolates. There were no profiles specific for human or animal isolates either, but specific electrophoretic patterns appeared in *T. mentagrophytes* isolated from both types of cases.

### Production of virulence factors and the host range of isolates

**Fig. 3:**
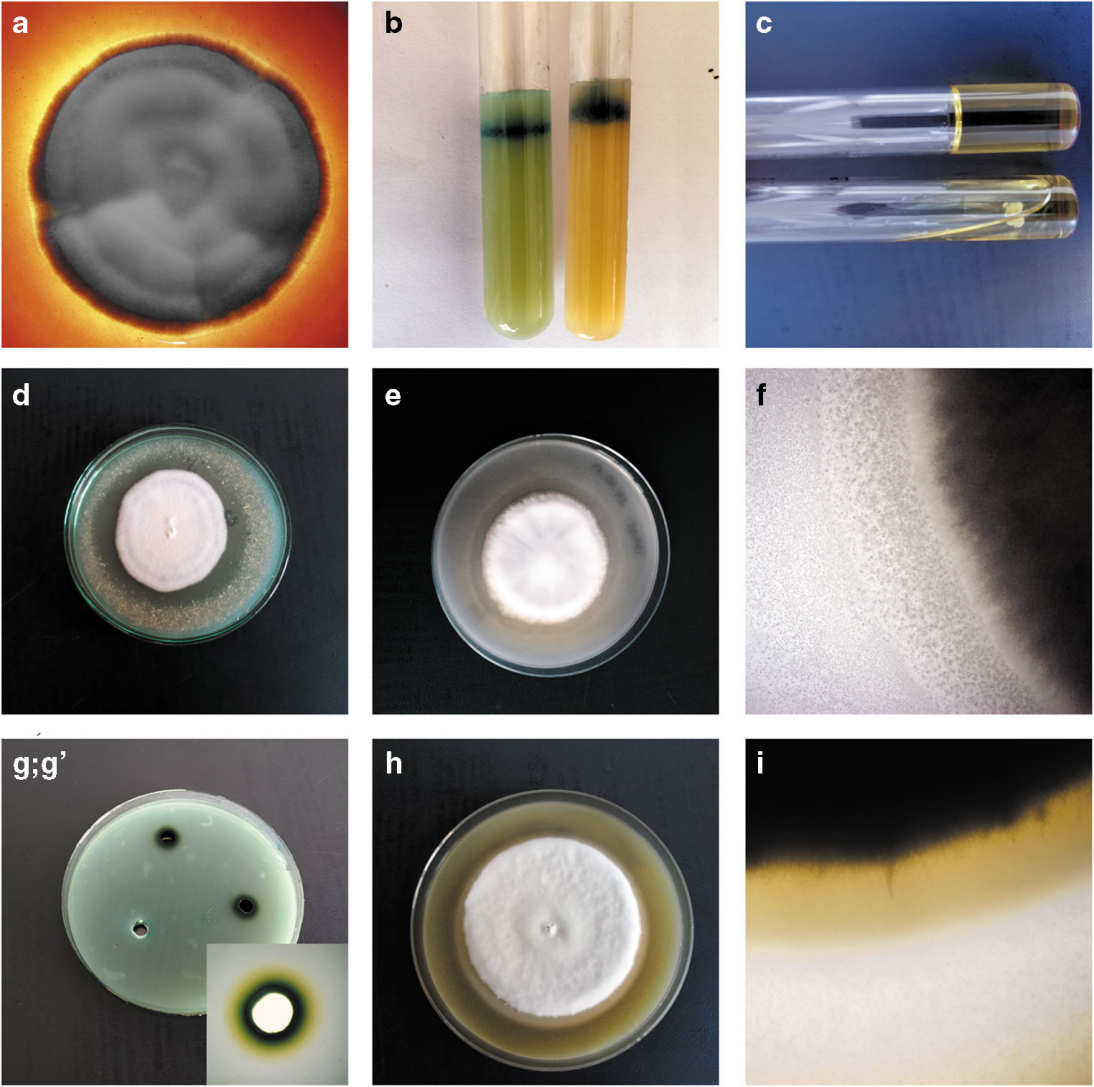
In vitro enzymatic activity of *Trichophyton mentagrophytes* strains obtained from human and animals cases. Notes: **a** – haemolytic activity; **b** – keratinase activity; **c** – gelatinase activity; **d** – elastase activity; **e**, **f** – lipase activity; **g**; g’ – catalase activity; **h**, **i** – phospholipase activity

The enzyme profiles obtained for the human and animal isolates were not significantly differentiated (Table 2). All the tested clinical isolates of *T. mentagrophytes* showed keratinase, phospholipase, protease, and catalase enzymatic activity as well as haemolytic activity (Fig. 3). In turn, extracellular elastase was produced only by the clinical isolates obtained from humans, and lipase was produced by the animal isolates. Furthermore, no strains diverging from the relationship were found: when the enzymatic activity was positive, it was exhibited by 100% of the tested isolates, as in the case of negative results.

**Table 2:** Enzymatic activity in vitro of *Trichophyton mentagrophytes* isolates obtained from humans and animals

Isolates	Keratinase	Phospholipase	Lipase	Protease	Elastase	Haemolysis	Catalase
Humans	+	+	−	+	+	+	+
Animals	+	+	+	+	−	+	+

**Fig. 4:**
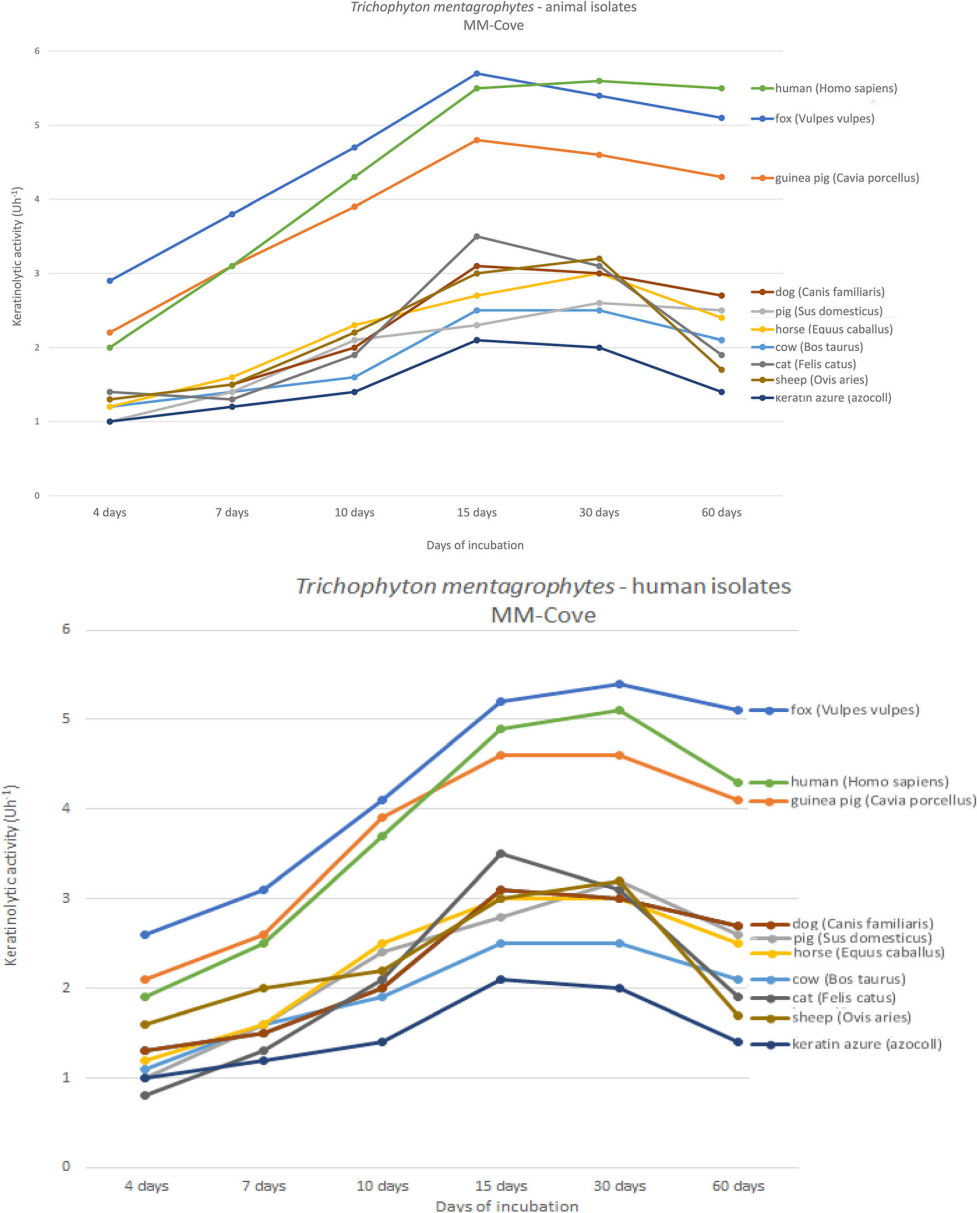
Graph of changes in keratinolytic activity (Uh^−1^) in various incubation periods on MM-Cove medium supplemented with species-specific types of keratins and keratin azure for the *Trichophyton mentagrophytes* clinical isolates

Particularly important is the species-specific keratinolytic activity, which is recognized as a determinant of the host range of dermatophytes. The degree of keratinolytic activity ranged from 0.8 Uh^−1^ and 1.0 Uh^−1^ on day 4 of incubation to 5.4 Uh^−1^ on day 30 of incubation and 5.8 Uh^−1^ on day 15 of incubation for the *T. mentagrophytes* clinical isolates from humans and animals, respectively (Fig. 4). The highest activity of keratinase enzymes of the tested isolates was recorded in media containing keratin from the fox (*Vulpes vulpes*), guinea pig (*Cavia porcellus*), and human (*Homo sapiens*) hairs. In these three cases, the degree of keratinolytic activity was statistically significantly higher (*p* < 0.05) than in the medium with keratin azure; it was 5.8 Uh^−1^, 5.7 Uh^−1^, and 4.9 Uh^−1^ for the fox, human, and guinea pig hairs on day 15 of incubation for the animal isolates, and 5.2 Uh^−1^, 4.95 Uh^−1^, and 4.6 Uh^−1^ for the human isolates, respectively. In turn, this value for keratin azure was 2.05 Uh^−1^. Additionally, the geometric mean of keratinolytic activity on day 15 of incubation for three most active species was statistically significantly higher than for all the other species-specific hairs, i.e. 5.46 Uh^−1^ vs. 3.13 Uh^−1^ and 4.92 Uh^−1^ vs. 2.98 Uh^−1^ for the animal and human isolates, respectively. Interestingly, the animal *T. mentagrophytes* isolates exhibited higher keratinolytic activity against human hairs than all the others on day 30 of incubation. On the contrary, this relationship was not revealed for the human isolates, where the keratinolytic activity was at the highest level for the fox keratin in all periods.

### Antifungal susceptibility tests

The sensitivity to the antifungal substances was similar in the clinical isolates of *T. mentagrophytes* obtained from humans and animals. The MIC ranges, geometric means of MICs, MIC_50_, MIC_90_, and mode ratios of the 12 antifungal drugs used are summarized in Table 3. Phenyl morpholine derivatives, i.e. amorolfine, exhibited superior activity against strains obtained from both humans and animals with the lowest MIC_50_ values. In turn, griseofulvin was found to exert the weakest in vitro effect and had the highest MIC_50_ values in the tested isolates. Additionally, fluconazole had the widest MIC range, i.e. 0.06–32 μg/ml for the human isolates and 0.125–32 μg/ml for the animal isolates, respectively. Remarkably, MIC_90_ values above 1 μg/ml were obtained for grisofulvin, enilconazole, ketoconazole, and fluconazole in both groups of the tested strains and for terbinafine and itraconazole in the case of the human *T. mentagrophytes* isolates. Interestingly, the lowest statistically significant geometric mean of the MIC values (*p* < 0.05) for the dermatophytes isolated from humans were noted for amorolfine. Furthermore, the lowest geometric mean MIC value (without statistical significance) was obtained for naftifine in the case of the animal isolates.

**Table 3:** In vitro antifungal susceptibilities of clinical isolates of *Trichophyton mentagrophytes* obtained from human and animal dermatophytosis

Antifungal agents		Host	MIC (μg/ml)														MIC range	MIC_50_	MIC_90_	MIC_GM_	Mode
			0.004	0.008	0.016	0.03	0.06	0.125	0.25	0.5	1	2	4	8	16	32					
Allylamine	NFT	Humans		4			3		10								0.008–0.25	0.25	0.25	0.16	0.25
		Animals		12	8	7											0.008–0.03	0.016	0.03	0.016^ST^	0.008
	TRB	Humans	6							1 (1)	5 (3)	5 (5)					0.004–2	1	2	0.91	0.004
		Animals	6	8	9	4 (1)											0.004–0.03	0.016	0.03	0.02	0.016
Polyenes	AMB	Humans					3	5	8	1							0.06–0.5	0.25	0.25	0.19	0.25
		Animals			4	9	7	7									0.016–0.125	0.06	0.125	0.06	0.03
	GRE	Humans							1	4	7	5					0.25–2	1	2	1.13	1
		Animals						2	5	5	7	8 (2)	3 (3)				0.125–4	1	2	1.44	2
Imidazoles	KTC	Humans					3	5		4	2 (1)	3 (2)					0.06–2	0.5	2	0.64	0.125
		Animals					2	3	3	12	7						0.06–1	0.5	1	0.53	0.5
	MCZ	Humans			3	4	6	4									0.016–0.125	0.06	0.125	0.06	0.06
		Animals		5	5	7	8	2									0.008–0.125	0.03	0.06	0.04	0.06
	ENC	Humans								9	5	3					0.5–2	0.5	2	0.91	0.5
		Animals				2	3	4	6			4	8				0.03–4	0.25	4	1.56	4
Triazoles	ITC	Humans					1	2	6	3 (1)	3 (1)	2 (1)					0.06–2	0.25	1	0.61	0.25
		Animals		5	5	5	7 (3)	2 (2)	2 (2)	1 (1)							0.008–0.5	0.03	0.125	0.07	0.06
	FLC	Humans					1	5	4	3	1			2 (1)		1 (1)	0.06–32	0.25	8	3.07	0.125
		Animals						1	3	4	4	5		1	7	2	0.125–32	2	16	7.44	16
	VRC	Humans		4	6	7											0.008–0.03	0.016	0.03	0.02	0.03
		Animals		2	4	7	8	3	3								0.008–0.25	0.06	0.125	0.07	0.06
Pyridinone derivatives	CPO	Humans	1	2		4	9	1									0.004–0.125	0.06	0.06	0.05	0.06
		Animals		3	9	8	4	3									0.008–0.125	0.03	0.06	0.04	0.016
Phenyl morpholine derivatives	AMR	Humans	6	9	1	1											0.004–0.03	0.008^ST^	0.008^ST^	0.008^ST^	0.008
		Animals	7	9	7	2	1	1									0.004–0.125	0.008^ST^	0.03	0.02	0.008

*AMB* amphotericin B, *AMR* amorolfine, *CPO* ciclopirox, *ENC* enilconazole, *FLC* fluconazole, *GRE* griseofulvin, *ITC* itraconazole, *KTC* ketoconazole, *MCZ* miconazole, *NFT* naftifine, *TRB* terbinafine, *VRC* voriconazole; (*n*) number of strains derived from patients treated orally with this substance; ^ST^statistically significantly the lowest result in human/animals group

## Discussion

Given the growing prevalence of superficial dermatophyte infections, especially in immunocompromised patients, these diseases are regarded as a public health issue worldwide [8]. The immune status of the host has been referred to as the main factor determining the outcome of the courses of the disease, which may range from limited cutaneous or subcutaneous infections to invasive disseminated life-threatening symptoms [45]. Despite their availability, the arsenal of antifungal drugs for clinical use acts on a limited number of cellular targets [46]. Moreover, the overlapping mechanisms of action of the commonly used drugs may contribute to emergence of multidrug resistance (MDR) phenotypes observed for several pathogenic fungi [47]. Additionally, it is common for a large group of patients and animal breeders to neglect and abandon treatment due to its cost, duration, and many side effects [48]. In this study, we present the characteristics of *T. mentagrophytes* dermatophytes obtained from patients and animals undergoing antifungal therapy (Table 1). Among the positive tests in 24 patients and 35 animals of different species selected by the real-time PCR technique, dermatophyte cultures were obtained in 17 and 27 cases, respectively. This represents a very high percentage (70.8% and 77.1%), indicating that living elements of the fungus are still present in the affected areas despite the treatment.

Molecular typing methods can provide crucial insights into the epidemiology and pathogenicity of dermatophytes [49, 50]. These techniques can also help to characterize infecting strains and monitor their occurrence and distribution [31]. Moreover, the most important investigation in the molecular epidemiology of dermatophytes is to determine whether infections are caused by the same or different strains [51]. In this aspect, disclosure of infection sources and transmission pathways in populations of humans and animals is necessary, and available techniques should allow deep genetic differentiation of strains within species, thus facilitating prompt and reliable identification of individual clones [52]. Our investigation showed a relatively high genomic diversity revealed by the MP-PCR analysis of clinical isolates of *T. mentagrophytes* of both human and animal origin. Although the MP-PCR method is widely described in the literature as a useful tool for the epidemiological analysis of the source of infection [31, 49, 51, 53], it seems that it cannot be used to detect recalcitrant to treatment dermatophyte isolates. The molecular basis of terbinafine resistance is most widely described to result mostly from changes at the genome level [18, 27, 54, 55]. However, in our study, out of 8 strains with in vitro resistance to this drug (MIC ≥1 μg/ml) obtained from patients treated with this substance, 4 different electrophoretic MP-PCR profiles were revealed. Thus, it is probably not possible to indicate one MP-PCR profile for *T. mentagrophytes* isolates exhibiting terbinafine resistance. There are no similar results in the literature and therefore this aspect requires more extensive analysis.

Despite the superficial localization of dermatophyte colonization, the host-fungus relationship in these infections is complex and not fully elucidated [22, 56]. Additionally, the pathophysiological mechanism is strictly correlated with the dermatophyte species, the host, and their immune status [57, 58]. Remarkably, the pattern of enzymes secreted by dermatophytes may underlie their survival in the host stratum corneum and, consequently, in the clinical pictures of the infection, not only by providing nutrients to the detriment of the keratinized barrier, but also by triggering and modulating the immune response [26, 40, 59]. The knowledge about the range of enzymes produced by dermatophytes with functions in pathogenesis is constantly growing; however, it is still not entirely clear whether the enzyme profile is the most important factor in the severity of symptoms [22, 60]. The data presented in this article show that dermatophytes isolated from animals and humans with skin lesions are able to produce different enzymes in vitro. However, it is difficult to capture the clear host-related relationship and the enzyme that causes recalcitrance to treatment. All analysed isolates produced keratinases, which are used by most dermatophytes to establish infection on hosts [44, 49]. However, as suggested by Mignon et al. [61] and Cafarchia et al. [62], it seems that keratinase activity is not associated with the presence of cutaneous lesions or any particular clinical picture of dermatophytosis. In contrast, the level of the activity of this enzyme might be correlated with the symptomatic infections of animals and humans, as shown in our research.

Furthermore, a distinct tendency indicating the highest keratinolytic activity of the *T. mentagrophytes* strains in the 15–30-day incubation period was revealed in our study. Wawrzkiewicz et al. [63] suggest that the keratinolytic activity of dermatophyte strains is connected with the fungal cell, and the enzyme is produced extracellularly only in the case of *T. verrucosum* strains. Thus, keratinolytic activity can be directly linked to the presence of dermatophyte mycelium, and its increase is associated with stronger pathogenicity [47, 64]. Additionally, another issue is the induction of the activity with a suitable substrate rather than the amount of enzyme protein in the culture [44, 65]. Our results indicate that the activity of *T. mentagrophytes* keratinase is induced by the substrate and the host range can be clearly determined (Fig. 4). This finding is in agreement with a study conducted by Mercer et al. [66]. The researchers conclude that the accumulation of keratinase does not correlate positively with higher intensity of natural keratin degradation, and the predisposition of enzymes resulting from the adaptation of the fungus to the natural host may play a key role. This dependence is noticeable in our studies. The clinical isolates of *T. mentagrophytes* showed higher in vitro keratinolytic activity against the fox, guinea pig, and human hairs than against the other ones. Initially, these observations were considered to indicate a source of fungal infection in humans, which was related to the high keratinolytic activity only for species-specific types of substrate [32, 67, 68]. Contrarily, the range of dermatophyte hosts can be closely correlated with the similar structure of keratin in the hair of these species [44, 69]. Final conclusions require more extensive research.

In the last decades, various new antifungal drugs with increased efficacy and an associated anti-inflammatory effect have been introduced and have broadened the munition against dermatophytosis [11, 70]. However, the treatment of this disease is still less successful than that of bacterial infections, especially because fungal cells are eukaryotic and much more similar to human and animal cells than bacteria [2]. Furthermore, recalcitrant dermatophyte infections may be related to inadequate or discontinued treatment, difficulties in eliminating predisposing factors in hosts or infection sources, and re-infections [17, 22, 49, 71]. According to experts, the minimum duration of therapy in recalcitrant cases of dermatophytoses should be 4 weeks [72]. However, there is no single official position of dermatologists on this subject and individual studies differ in interpretations. Nonetheless, recalcitrant or recurrent infections after completion of a recommended therapy or antifungal drug-resistant dermatophytes are well known to dermatologist and veterinarians. In addition, scientific literature suggests that drug resistance is on the rise in dermatophytes, although correlation between in vitro resistance and therapeutic failure is noted in a very small number of cases [19, 27, 46, 73–75]. The cases of dermatophytoses in humans and animals described in this study were recalcitrant to treatment. We employed the broth microdilution methodologies using the CLSI M38 [76] standard to determine the MICs of the antifungal agents for the tested *T. mentagrophytes* clinical isolates. Our results indicate that the in vitro antifungal activity of the drug used in oral therapy were above 1 μg/ml in 15 (82.8%) and 5 (35.7%) cases of the human and animal infections, respectively. The cutoff value of MIC equal or higher than 1 μg/ml is considered in many scientific reports as an indicator of dermatophyte resistance to a given substance [17–19, 77]. Nonetheless, the term “resistance” and the breakpoint of 1 μg/ml for dermatophytes need an appropriate context and the limitation of wide use is the lack of a clear link to clinical failure of treatment. Indira [78], Bhatia and Sharma [79], and Poojary [80] reported that MIC_90_ ranges for griseofulvin, itraconazole, and fluconazole were significantly higher against *T. mentagrophytes* isolates than against *T. rubrum*. Generally, Tamura et al. [81] suggested that *T. mentagrophytes* strains were more resistant to azoles than *T. rubrum* and the MIC ranges of the non-azole agents, i.e. amorolfine or terbinafine and butenafine, against *Trichophyton* spp. were relatively narrow compared to those of azole agents. However, increasing numbers of cases from Asian and European countries can be found in literature reports on dermatophytoses that are difficult to treat with terbinafine, which indicates that microbial resistance to this substance is on the rise [27, 54, 55, 82–86]. Similarly, recent reports in literature have revealed that the trend of increasing MIC values for terbinafine in the *T. mentagrophytes* isolates is observed over the years. In the years 2009 to 2012, the MIC_50_ for this drug was determined in the range of 0.06–0.125 μg/ml [87–90], and in 2018, this value increased up to 1 μg/ml [27]. Clinical evidence of relapse and incomplete mycological cure after standard oral terbinafine therapy, i.e. 250 mg, twice daily for 2 weeks have also been reported [91]. Sakai et al. [92] showed that the use of 250 mg of terbinafine twice daily was appropriate for treatment of dermatophyte infections caused by *T. mentagrophytes* of animal origin with a MIC of 0.01 μg/ml. In the present study, we observed that approximately 65% of human and 48% of animal isolates had a terbinafine MIC higher than 0.01 μg/ml. Furthermore, the tissues infected by dermatophytes are avascular components of the skin; the time to attain therapeutic concentrations in them may differ greatly from plasma [92, 93]. In consequence, a longer therapy strategy may be required to treat infections by *T. mentagrophytes* isolates with higher MICs. Unfortunately, this may not be clinically practical due to the possibility of drug-related side effects. Therefore, the choice of a proper drug for the therapy of dermatophyte infections is increasingly complicated and requires extensive knowledge. Our results indicate that there is no one-size-fits-all treatment pattern and no ideal antifungal substance, and the difficulties in therapy can be directly related to drug resistance in dermatophytes.

Interestingly, resistance to antifungal drugs seems to be of much less importance in connection with the failure of therapy in animals than in humans. This may be correlated with the frequently noted status of an animal asymptomatic carrier of dermatophytes [49, 53, 94]. Symptoms of infections in these animals can be observed only in certain host immune deficiency states, which are a major factor in subsequent treatment failures [32, 95]. Moreover, more intensive contact of animals with soil may favour the easy acquisition of infectious elements of dermatophytes, for which soil is one of the most important reservoirs [75, 96–98].

Finally, the difficulties in treating dermatophytoses may have a variety of causes that are not always related to the pathogen but result from the immunology of the host and his lifestyle. The increased frequency of reported refractory dermatophyte infections is now becoming a public health problem and the search for its key causes is necessary for new therapeutic approaches. Analysis of a large group of clinical isolates obtained from humans and animals with long-lasting dermatophytoses indicates that fungal drug resistance is increasing. The causes of recalcitrant cases should be sought mainly in this phenomenon, and monitoring the susceptibility to antifungal drugs should be almost a routine examination at every emerging outbreak of the disease.

## Data Availability

All data generated or analysed during this study are included in this published article. Detailed data are available from the correspondence authors on request.
